# Quorum sensing-related activities of beneficial and pathogenic bacteria have important implications for plant and human health

**DOI:** 10.1093/femsec/fiae076

**Published:** 2024-05-14

**Authors:** Anton Hartmann, Tatiana Binder, Michael Rothballer

**Affiliations:** Faculty of Biology, Microbe-Host Interactions, Ludwig-Maximilian-University Munich, Grosshaderner Str. 2, D-82152 Planegg/Martinsried, Germany; Department of Environmental Sciences, Helmholtz Zentrum Munich, German Research Center for Health and Environment, Research Unit Microbe-Plant Interactions, Ingolstädter Landstr. 1, D-85762 Neuherberg, Germany; Department of Environmental Sciences, Helmholtz Zentrum Munich, German Research Center for Health and Environment, Research Unit Microbe-Plant Interactions, Ingolstädter Landstr. 1, D-85762 Neuherberg, Germany; Department of Environmental Sciences, Helmholtz Zentrum Munich, German Research Center for Health and Environment, Research Unit Microbe-Plant Interactions, Ingolstädter Landstr. 1, D-85762 Neuherberg, Germany; Helmholtz Zentrum Munich, German Research Center for Health and Environment, Institute of Network Biology, Ingolstädter Landstr. 1 D-85762 Neuherberg, Germany

**Keywords:** control of pathogens, host beneficial microbes, *N*-acyl-homoserine lactones, One Health concept, opportunistic human pathogens, quorum sensing molecules, systemic induction of tolerance to abiotic stress

## Abstract

Eukaryotic organisms coevolved with microbes from the environment forming holobiotic meta-genomic units. Members of host-associated microbiomes have commensalic, beneficial/symbiotic, or pathogenic phenotypes. More than 100 years ago, Lorenz Hiltner, pioneer of soil microbiology, introduced the term ‘Rhizosphere’ to characterize the observation that a high density of saprophytic, beneficial, and pathogenic microbes are attracted by root exudates. The balance between these types of microbes decide about the health of the host. Nowadays we know, that for the interaction of microbes with all eukaryotic hosts similar principles and processes of cooperative and competitive functions are in action. Small diffusible molecules like (phyto)hormones, volatiles and quorum sensing signals are examples for mediators of interspecies and cross-kingdom interactions. Quorum sensing of bacteria is mediated by different autoinducible metabolites in a density-dependent manner. In this perspective publication, the role of QS-related activities for the health of hosts will be discussed focussing mostly on *N*-acyl-homoserine lactones (AHL). It is also considered that in some cases very close phylogenetic relations exist between plant beneficial and opportunistic human pathogenic bacteria. Based on a genome and system-targeted new understanding, sociomicrobiological solutions are possible for the biocontrol of diseases and the health improvement of eukaryotic hosts.

## Teaser

Quorum sensing-related activities of beneficial and pathogenic bacteria associated with plant and human have important implications for host health and well-being.

## Introduction

As result of the evolution of life on Earth, all higher organisms are living as holobiots characterized by tight interaction with a diversity of microbes. The hologenome concept considers holobionts as units of coevolution and selection of well-adapted pro- and eukaryotic organisms leading to constructive co-operations (Stencel et al. 2018). In the evolution of land plants, soil microbes found roots as most attractive interaction area. More than 100 years ago, the term ‘Rhizosphere’ was defined by Professor Lorenz Hiltner as the plant root-dominated habitat where soil microbes live on the expense of low and high molecular weight root exudates (Hiltner 1904, Hartmann et al. 2008). Symbiotic and plant-beneficial interactions evolved in specific bacteria and fungi contributing to major needs of the plant, such as the supply with essential nutrients like nitrogen from the air and macro- and micronutrients from the soil. Nitrogen-fixing bacteria or mycorrhizal fungi are prominent examples for plant-symbiotic microbes. As already Lorenz Hiltner recognized, not only saprophytic and beneficial but also pathogenic microbes are attracted by root exudates, causing chances and challenges for plant health (Mendes et al. 2013, Abedini et al. 2021). Most recently, rhizosphere phage communities were identified to suppress bacterial plant disease (Yang et al. 2023). Mechanisms have been identified by which plants can form the rhizosphere microbiome in a kind of ‘rhizosphere school’ to support healthy plant development (Berendsen et al. 2012). Biofilms are the natural habitats for microbes, and therefore cooperative and competitive interactions within complex surface microbial biofilms are of key importance during root colonization (Nadell et al. 2016). These interactions decide about success or failure of beneficial microbes to support plant health or of pathogens to develop diseases. In the densely populated biofilms of the rhizosphere, bacteria optimize the expression of their genetic potential using diffusible signal molecules to sense the density of their own and neighbouring population (Fuqua et al. 1994). Quorum sensing (QS) is of central importance for successful adaptation to changing environmental conditions and colonization of and eventual establishment within an eucaryotic host (Mukherjee and Bassler 2019). The rhizosphere also provides a stage for multiple avenues of natural genetic engineering, involving horizontal gene transfer (Van Elsas et al. 2003), transduction of genetic elements with plasmids (Shintani et al. 2020), enhanced mutation rates, and phenotypic variations (Achouak et al. 2004, van de Broek et al. 2005, Li et al. 2012, Lalaouna et al 2011). For example, many bacteria acquired multiple *luxI-* and/or *luxR-*QS gene homologues from independent sources via horizontal gene transfer (Lerat and Moran 2004). QS activities are prevalent in commensalic, beneficial/symbiotic, and pathogenic plant-associated bacteria (von Bodman et al. 2003).

**Figure 1.**
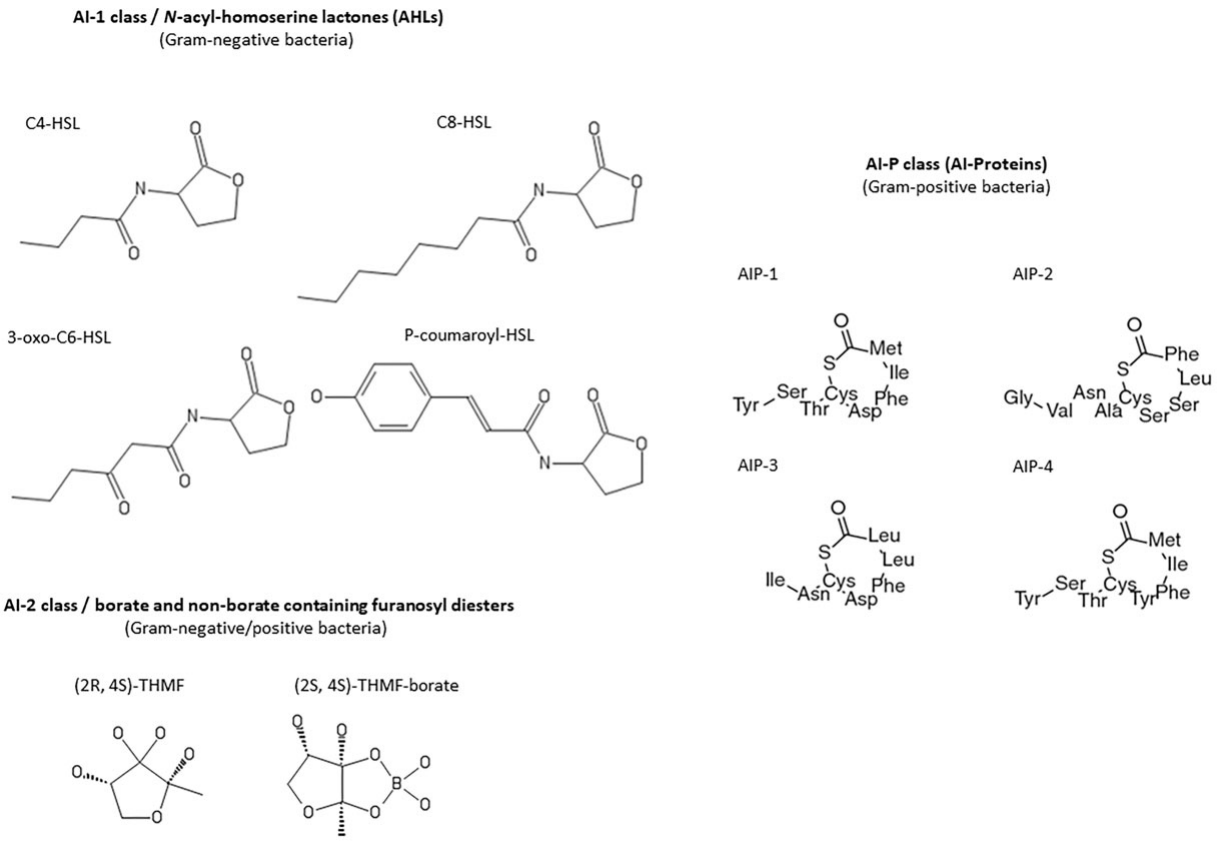
Structures of QS autoinducers: AI-1 (AHLs), AI-2 (furanosyl borate diester), and AI-P (autoinducer peptide): In AI-1/AHLs, the C3-carbon of the carbonyl fatty acid chain can alternatively carry a hydroxy-residue; the fatty acid chain may also carry a C–C double bond. AI-2 can exist as the boron containing S-THMF-borate (2S, 4S)-2-methyl-2,3,3,4-tetrahydroxytetrahydrofuran (QS-active form) or the nonboron form R-THMF (2R, 4S)-2-methyl-2,3,3,4-tetrahydroxytetrahydrofuran. The AI-P1, AI-P2, AI-P3, and AI-P4 structures were identified in *S. aureus* (Schaefer et al. 2008).

The first evidence for the involvement of QS-signals and their perceptions by plant hosts were revealed by Mathesius et al. (2003) analysing the response to AHL-QS-signals by *Medicago truncatula* and by Schuhegger et al. (2006) studying induction of pathogen resistance after AHL-application to roots of tomato plants. Furthermore, QS-related genes were identified in the endosphere bacterial metagenome of rice by Sessitsch et al. (2012) and in the bacterial microbiome of *Populus deltoides* (Schaefer et al. 2013). In addition, bacteria harbouring QS-genes were frequently isolated from the rhizosphere and endosphere of plants. The ability to communicate with each other and to trigger essential functions in a timely and optimized manner is an important feature for single cell organisms to efficiently use their genetic potential as an organized community (Hense et al. 2007). Among the QS systems, *N-*acyl-homoserine lactone (AHL, AI-1) circuits are present in many Gram-negative bacteria (Fuqua et al. 1994). AHLs are small diffusable molecules having different structures consisting of a fatty acid moiety with different chain length (4–20), and l-homoserine lactone (Fig. 1). The fatty acid chain can be modified at the C3-position with a hydroxy- or oxogroup or replaced by a *p*-coumaroyl- or cinnamon-residue (Schaefer et al. 2008, Ahlgree et al. 2011). AHL-biosynthesis and perception occurs through a two-component LuxI/LuxR system. The LuxI-enzyme catalyses the binding of acylcarrier protein-bound *S*-adenosylmethionine to the acyl chains resulting in AHLs. The LuxR-receptor binds AHLs and the LuxR-AHL-complex finally activates diverse promotors, including the *LuxI-*promotor. The special feature of QS-signalling is that small and diffusible molecules are constitutively produced in very low amounts. When AHL-concentrations rise in more dense populations above a critical concentration, called quorum, its biosynthesis is autoinduced. At high signal concentrations, a set of genes (the QS-activated transcriptomes) is induced by the LuxR–AHL complex. This opens up new areas of functions essential, e.g. for efficient colonization of a new host. QS-controlled genes often code for the construction of biofilms, hydrolysis and degradation of nutritional carbon polymers and substances, the induction of virulence, and the synthesis of diverse siderophores and antibiotics (Hense and Schuster 2015). The so-called autoinducer-2 (AI-2), furanosyl borate diester, are produced by some Gram-negative and Gram-positive bacteria (Papenfort and Bassler 2016) (Fig. 1). AI-2 is synthesized by the LuxS-protein, and specific AI-2 receptors (e.g. LuxPQ-receptor protein of *Vibrio harveyi*). The lack of genomic evidence of AI-2 receptors in some bacteria may suggest a non-QS role for LuxS in these bacteria (Rezzonico and Duffy 2008). In Gram-positive bacteria, like *Streptococcus pneumoniae* or *Staphylococcus aureus*, different autoinducing peptides (AI-P) are produced (Fig. 1), which activate e.g. virulence and toxin production during growth in biofilms in a density-dependent manner (Parsek and Greenberg 2005). Further QS-signal molecules are mentioned by Hartmann et al. (2021). Besides QS-signals, root-associated microbes produce a high diversity of signalling compounds, like volatiles (Schultz-Bohm et al. 2017, de Boer et al. 2019), and diverse plant hormones, which influence plant hosts in multiple ways (Gamalero et al. 2023).

Since microbes interact with all eukaryotes in holobiotic-type life forms, comparable signalling mechanisms and principles of cooperation and/or competition are present in all holobionts, which will be discussed in this perspective publication. Likewise, different organs of mammalian hosts, including human, like skin, oral cavity, lung, or gut are colonized with QS-active bacteria of various types, which have beneficial or pathogenic features (Whiteley et al. 2017). It also needs to be considered that in some cases very close phylogenetic relationships exist between plant beneficial and opportunistic human pathogenic bacteria (Berg et al. 2005, 2014, Abreo and Altier 2019, Faoro et al. 2019). In the translation of potentially therapeutic or supportive approaches for sustainable agriculture or human health, the application of metabolites or probiotic microbes with health-supporting signalling capabilities could be of central importance to better control pathogens and support health and well-being of eukaryotic hosts based on natural socio-microbiological mechanisms.

### QS-AHL signalling: different AHL functions in plants, LuxR solo receptors, QQ activities, AHL uptake, and signal perception

Many Gram-negative pathogenic bacteria organize their virulence using the AHL-circuit to attack plants (Pollumaa et al. 2012), while symbiotic nitrogen-fixing rhizobia initiate and coordinate root colonization and nodule formation with a diversity of AHL-autoinducers (González and Marketon 2003). The importance and wide distribution of QS-signals in bacteria–plant interaction provide the coevolutionary rational for the sensitive recognition and perception of AHLs by plants leading to an effective stimulation of the innate immune defence system also by beneficial rhizosphere bacteria initiating specific pathogen defence measures or improving abiotic stress tolerance (Schikora et al. 2016). The AHL-based priming of defence is an cost-efficient strategy to fight back pathogens (Shrestha et al. 2020).

### LuxR-solo receptors

In addition to the cognate LuxI/LuxR-signal circuit, many Gram-negative bacteria harbour one or several so-called LuxR solos, which are not paired with a classical LuxI-type synthetase, and are thus unable to produce AHLs themselves (González and Venturi 2013). These luxR-solos play a pivotal role in intra- and interspecies, as well as interkingdom, communication. Different classes of LuxR-solo systems can be distinguished, which perceive endogenous and exogenuos AHLs as well as non-AHLs signals (Bez et al. 2023). LuxR regulators are widely distributed bacterial helix-turn-helix transcription factors involved in QS-type mechanisms. They are also found in 50% of the genomes of Gram-positive Actinobacteria for traits at environmental and medical levels in connection with QS and virulence strategies (Santos et al. 2012, Sarveswari and Solomon 2019). As example for non-AHL exogenous signals, host-specific signals of kiwifruit are perceived by PsaR2 LuxR solo in *Pseudomonas syringae* pv. *actinidiae*, aimed to induce virulence factors like biofilm formation, motility and endophytic colonization (Cellini et al. 2022). This subclass of LuxR solos is frequently found in plant-associated bacteria, both beneficial/symbiotic or pathogenic bacteria responding to different plant signals (Patel et al. 2013). In some cases the signals are similar to AHLs; e.g. BraR from the stem-nodulating legume symbiont *Bradyrhizobium japonicum* responds to cinnamoyl-homoserine-HSL, derived from the plant metabolite cinnamon (Ahlgreen et al. 2011). RpaR from *Rhodopseudomonas palustris* binds to *p*-coumaroyl-HSL derived from the exogenous *p*-coumarate (Schaefer et al. 2008). The LuxR-solo QscB receptor and the QscR regulon in the pathogen *P. aeruginosa* is different from the also present canonical LuxI/LuxR tandem and stimulates virulence activities through increased biosynthesis of the antibiotic phenazine (Fuqua et al. 1994).

### QQ activities by AHL hydrolysis, oxidoreductase, and other quenching mechanisms

In the light of the central role of QS for virulence acquisition, the inhibition of QS, including quorum quenching (QQ) by cleaving and inactivating the QS-moieties or by inhibiting the QS-autoinducer action is attracting high attention. Inhibitory mechanisms include blocking the QS-signal synthesis, inhibition of the autoinducer reception, and signal transport (summarized by Hartmann et al. 2021). A large number of especially Gram-positive bacteria and even fungi, but also plant and mammalian hosts, harbour different types of AHL-quenching activities (Grandclément et al. 2016). AHL-signals can undergo different modes of degradation and inactivation in the rhizosphere. Chemical hydrolysis of AHLs occurs at neutral and alkalic pH-values but AHLs are stable at acid pH-values. Efficient enzymatic degradation occurs through AHL-lactonases and AHL-acylases/amidases. In addition, AHL-oxido-reductases were described (Chowdhary et al. 2007). Growth and QQ-activity of *Rhodococcus erythropolis* R138 was efficiently stimulated by the organic amendment of gammaheptalactone leading to efficient biocontrol of potato (Cirou et al. 2012). Also, a strong inhibition of the QS-regulated pathogen *Pectobacterium atrosepticum* was shown by gamma-lactone stimulated *R. erythropolis* resulting in efficient *in planta* biocontrol (Barbey et al. 2013). Furthermore, a novel type of AHL-acylase of *Ochrobactrum* sp. A44 was demonstrated to quench the AHL-dependent virulence of *P. carotovorum in planta* (Czajkowski et al. 2011). In the context of coral reef disease—a case of high global importance—the application of QS-antagonists to white band disease-infected *Acropora cervicornis* inhibited disease-causing bacteria and stopped coral reef disease development (Certner and Vollmer 2018). Most recently, a novel type IVA secretion system (T4ASS) effector was discovered in *Lysobacter enzymogenes* OH11 (Liao et al. 2023). This T4ASS is able to deliver a protein (Le1288) into *P. fluorescens* SPL4, which acts there as a AHL-synthase inhibitor. It was shown that this T4ASS-mechanism is working also to inhibit the human pathogen *P. aeruginosa* and the plant pathogen *Ralstonia solanacearum* (Liao et al. 2023). QS-related interventive actions within host-associated microbiomes are of high general relevance also for the balance of beneficial and pathogenic bacteria in mammalian and human health (see below). In *Arabidopsis thaliana* and some other dicotyledonous plants, especially legumes, fatty acid amid hydrolases cleave AHLs by liberating the fatty acid tail and l-homoserine (HS; Palmer et al. 2014). Interestingly, l-HS is not only able to stimulate root growth but also improve water and nutrient uptake into the plant (Palmer et al. 2014). It was even found that AHL-producing rhizobacteria, like *Pseudomonas putida* IsoF, itself harbour AHL-degrading activities, leading to reduced intra- and extracellular AHL-concentrations (Fekete et al. 2010). The biosynthesis of C10-HSL in batch as well as contiuous cultures of *P. putida* IsoF and the simultaneous appearance of the cleavage product l-HS were quantified using high resolving UPLC-measurements and ELISA-technique (Chen et al. 2010, Buddrus-Schiemann et al. 2014). The ecological meaning of this activity could be to limit the AHL-production to a specific culture phase. Alternatively, it could provide plants in another growth phase with the stimulatory l-HS (Palmer et al. 2014).

### AHL-uptake

Some plants, like wheat and barley, apparently lack AHL-degrading enzymes and take up unhydrolyzed AHLs. Water soluble C6–C10 AHLs were shown to be taken up into the shoot via active transport (Götz et al. 2007, Sieper et al. 2014). The transport inside the roots occurs in the central cylinder as was shown by autoradiography using ^3^H-labelled C8- and C10-HSL; the transport was inhibited by orthovanadate, demonstrating that ABC-transporters are involved. The identity of the transported AHLs in the shoots was proven and quantified by AHL-specific monoclonal antibodies, developed by Chen et al. (2010), and AHL-sensor strains (Sieper et al. 2014). In contrast to *Hordeum vulgare* (cv Barke), the legume *Pachyrhizum erosus* (L.) did not take up AHL efficiently, as was shown by ultra-performance liquid chromatography (UPLC) and Fourier transform ion cyclotron resonance (FTICR)-mass spectrometry (Götz et al. 2007), due to AHL-degradation in the roots. Further detailed analysis via FTICR-MS and UPLC revealed a metabolism towards C3-hydroxy- and C3-oxo-HSLs in the root compartment especially for C8- and C10-HSL, which may contribute to reduce the transport into the shoot (Götz-Rösch et al. 2015). In addition, a chiral separation of d/l-forms by GC–MS demonstrated that barley selects the l-forms during active transport (Götz et al. 2007, Sieper et al. 2014).

### AHL perception as plant growth stimulans and priming agent for pathogen and abiotic stress tolerance

**Figure 2.**
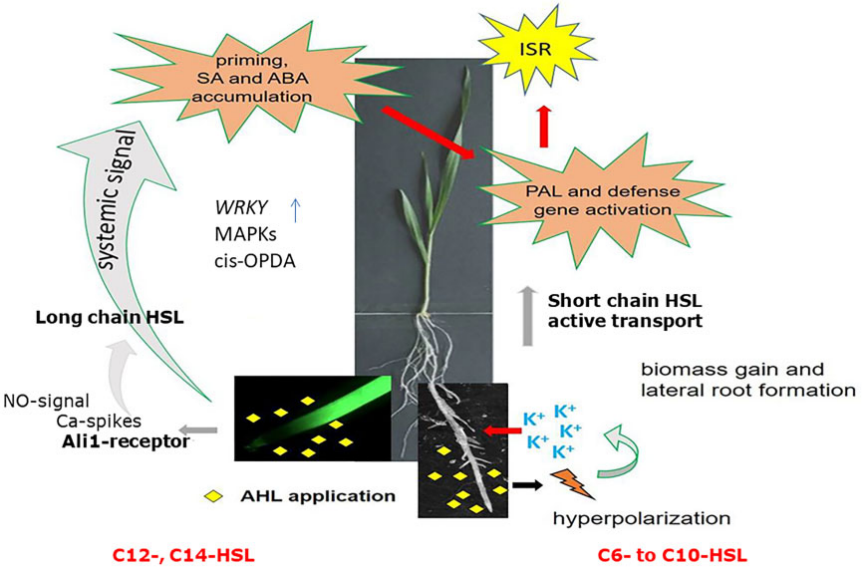
AHL interactions with plants (example: *Arabidopsis*, barley): the perception of AHLs by plants can be divided into interactions with short- and long-carbonyl side chain AHLs. Water-soluble AHLs (like C6- to C10-HSLs) are transported in an active transport process through the central cylinder (Sieper et al. 2014) to the shoot, if AHLs are not degraded by plant lactonases. In the roots, hyperpolarization and K^+^-uptake occurs and growth and lateral root formation is increased (von Rad et al. 2008, Liu et al. 2012, Rankl et al. 2016). In addition, abiotic and defence gene are activated in the shoots (Götz-Rösch et al. 2015). Lipophilic AHLs like C12- and C14-HSLs are perceived by a membrane protein (ALI1) (Shrestha et al. 2022). In the roots, NO is produced and a systemic signalling cascade to the shoots is activated including salicylic acid and oxylipin (cis-OPA/12-oxo-phytodienolic acid) leading to increased expression of MAP-kinases (MAKs) and defence-related transcription factors WRKY22 and 29 (Shrestha et al. 2019, 2020).

In barley, initial reactions occurs in root cells after treatment with 10 µM C6-, C8-, and C12-HSL (Rankl et al. 2016). Nitric oxide (NO) accumulates in the calyptra and root elongation zone and also the lateral root formation is changed. In addition, increased K^+^-uptake occurs in root cells, and membrane hyperpolarization is promoted in epidermal root cells especially by C8-HSL (Rankl et al. 2016). Upon application of C6-, C8-, and C10-HSL to the roots, the antioxidative and detoxifying capacities are increased in the shoots of barley (Fig. 2). As compared to control plants, the activity of dehydroascorbate reductase in barley shoots after C10-HSL treatment is greatly increased, whereas superoxide dismutase activity is slightly decreased after application of C6-HSL to the root system (Götz-Rösch et al. 2015). In contrast, the response of antioxidative enzymes in leaves of yam beans was low probably due to reduced uptake of AHLs. In addition, the response of cytosolic glutathion-S-transferase (GST) isoforms in roots and leaves to AHLs were increased or decreased dependent on the iso-form tested in root or shoot compartments in comparison with yam bean (Götz-Rösch et al. 2015). In the light of the observed responses of antioxidant and detoxifying plant activities towards AHLs, these QS-signals may be regarded as strengthening agents or plant antistress boosters.

The molecular structure of AHL-autoinducers determines the mode of action towards plant growth stimulation or priming of pathogen resistance (Fig. 2). For example in barley and wheat, which are devoid of AHL-lactonases and -hydrolases, water-soluble AHLs are taken up into the shoots by an energy-dependent transport leading to enzymatic changes in leaves (Sieper et al. 2014, Götz-Rösch et al. 2015). Water-soluble C4-, C6-, and C8 HSLs were also shown to change the phytohormone balance of *A. thaliana* seedlings, modifying root/shoot growth and metabolism (von Rad et al. 2008). When these AHLs were added to roots (at 10 µM concentration) a multitude of genes, mostly connected with phytohormone regulation, were newly induced in roots and shoots, while others were repressed (von Rad et al. 2008). A special role in this stimulation with C6- and C8-AHLs was identified for the GCR1/GPA1 genes in *A. thaliana* (Liu et al. 2012). While root growth of GCR1-mutants failed to be stimulated by C6- and C8-HSL, overexpressing mutants showed increased growth responses. In addition, calmodulin receptors were involved in primary root elongation, caused by 3-oxo-C6 HSL (Zhao et al. 2015). Furthermore, AtMYB44 was involved in enhanced elongation of the primary root by increasing cell divisions in the root meristem and enhanced cell elongation in the elongation zone (Zhao et al. 2016); this was accompanied by altering the cytokinin and auxin metabolism in roots. In *Arabidopsis* and wheat, 3-oxo-C6 HSL was able to enhance salt tolerant growth (Zhao et al. 2020). In 3-oxo-C6-treated salt-stressed plants, the content of chlorophyll as well as the osmolyte proline was increased and the content of malonedialdehyde and the Na^+^/K^+^-ratio were decreased (Zhao et al. 2020).

AHLs with long aliphatic chains (C12- and C14-HSL), which cannot be transported into the shoot, are also able to modify plants by conferring systemic resistance towards biotrophic and hemibiotrophic pathogens via altered activation of AtMPK6 (Schikora et al. 2011a). In barley, this response was only present in certain cultivars and therefore has to be regarded as a genetically determined property (Shesthra et al. 2019). In *Arabidopsis*, as early response specific receptors could be involved to perceive the AHL-signal and to activate a signalling cascade including salicylic acid (SA) and oxylipin 12-oxo-phytodienolic acid (cis-OPDA) (Schenk and Schikora 2015; Shrestha et al. 2020) (Fig. 2). In the presence of flagellin-derived peptide flg22 and C12-HSL, MAP-kinases MPK3, and MPK6 are increasingly stimulated and expressed for a prolonged time along with the upregulation of defence-related transcription factors WRKY22 and WRKY29 (Shrestha et al. 2019) (Fig. 2). Also, glutathione-S-transferase *GST6*-gene and the heat shock protein Hsp60 are induced. In *Arabidosis* exposed to mixtures of 3-oxo-C6-, 3-oxo-C8-, 3-oxo-C12-, and 3-oxo-C14-HSLs the response differ as compared to plants treated with single AHLs and jasmonates play an important role (Duan et al. 2023). The fast and stable decreased concentration of COOH-JA-Ile after challenge with flg22 as well as the JA- and SA-affected *Arabidopsis* mutants strengthened the conclusion that JA-homoeostasis is involved in AHL-priming (Duan et al. 2023). However, a deeper understanding of specific plant factors mediating the response to water-insoluble AHLs, like 3-oxo-C14-HSL, was missing. Most recently, the comparison of wild-type *A. thaliana* Col-0 and the oxo-C14-HSL insensitive mutant *ali1* allowed deeper insights. In *Arabidopsis*, the gene *AtGlcAk2* for glucuronokinase 2 is identical with *ali1* (Shrestha et al. 2022) (Fig. 2). Zhao et al. (2013) had described this gene already as a putative kinase from the GHMP kinase family being involved in root and flower development, abscisic acid signalling, and stress response influencing the expression of various ABA-related genes as well as salt stress. MAP-kinase activity measurements, gene expression, and transcriptome analyses as well as pathogenicity assays confirmed a loss of AHL-priming in *AtGlcAK2/Ali1* mutants (Shrestha et al. 2022). Furthermore, when fluorescently tagged ALI1-protein was expressed in tobacco leaves, ALI1 colocalized with the plasma membrane, tonoplast, and endoplasmic reticulum in the cell periphery. Thus, the ALI1-protein may be regarded as surface receptor for 3-oxo-C14-HSL and other water-insoluble AHLs. This novel insights may further improve the development of stress resistance of plants, useful for sustainable crop management (Shrestha et al. 2022).

Transgenically modified plants with introduced QS-autoinducer synthesis genes are able to communicate with bacteria in the rhizosphere by altering their QS-controlled activities (Fray et al. 1999). For example, tomato plants harbouring the AHL-biosynthesis genes *yenI* and *lasI* from *Burkholderia graminis* strains altered the activity of these strains in the rhizosphere, leading either to increased or decreased plant growth stimulation or resistance towards salt stress (Barriuso et al. 2008).

### QS-/AHL-systems in plant growth promoting and pathogenic bacteria

It has become apparent that within bacterial genera, which are known for efficient rhizosphere and endophytic root colonization, plant beneficial, symbiotic, and even opportunistic human pathogenic bacteria are closely related. Several examples are presented below, which demonstrate that plant beneficial bacteria with probiotic potential exist in the same species with opportunistic pathogens. Therefore, the need for careful evaluation of possible health risks for applications of these bacteria in agricultural or for biotechnological purposes is necessary, if the separation from pathogens are not approved by clear phylogenetic criteria.

A high diversity of AHL-producing, plant beneficial Gram-negative bacteria are now known since several decades to support growth of many crop plants under challenging abiotic and biotic conditions. *Gluconacetobacter diazotrophicus* PAL5 is an endophytic diazotrophic Gram-negative bacterium, first isolated in 1988 from inside sugar cane stems (Cavalcante and Döbereiner 1988). It can colonize numerous other plant species and confers several beneficial effects including abiotic and biotic stress tolerance and improved plant growth. In addition to the biological nitrogen-fixation activity and the production of several plant hormones, it was shown to harbour an active AHL-QS regulatory system (Bertini et al. 2014) producing eight different QS-signals based on C6-, C8-, C10-, C12-, and C14-HSL (Nieto-Penalver et al. 2012). When *G. diazotrophicus* PAL5 was inoculated to red rice plants growing under water stress conditions, the expression of the *LuxI*-gene was strongly stimulated at increasing water deficit conditions. The transcription of the PR1- and PR10-genes along with several antistress defence genes increased, like catalase and superoxide dismutase as well as ascorbate peroxidase (Filgueiras et al. 2020). The induced systemic tolerance to water deficit was accompanied by the accumulation of osmoprotectant solutes and the expression of defence genes against water deficit in plant shoots. Furthermore, mutations in the LuxI/R system of PAL5 resulted in strong reduction in endophytic root colonization of sugar cane seedlings (Hartmann et al. 2019).

Almost 50 years ago, diazotrophic bacteria species of the genus *Azospirillum* (*A. lipoferum* and *A. brasilense*) were isolated and characterized by the group of Johanna Döbereiner in Brazil (Döbereiner and Day 1976). Nowadays, at least 15 different species, mostly of root-associated plant growth-promoting bacteria are officially described within this genus, demonstrating the rich diversity of this efficient rhizosphere bacterium. Phytohormone interactions, based on indole acetic acid (IAA) production and other phytohormones by these bacteria, are prevalent for successful interaction with diverse plants, like wheat, maize, sorghum, causing multiple plant growth-stimulating effects. Although, *LuxI*-genes and the productions of AHLs were only found in some strains of *A. lipoferum* (Vial et al. 2006), the addition of C6- and C8-AHLs could stimulate biofilm formation, EPS-production and mobility in strain *A. brasilense* Ab-V5, a successfully applied *A. brasilense* inoculant strain (Fukami et al. 2018). It turned out that many *Azospirillum* spp. strains harbour multiple copies of *luxR*, so-called LuxR-solo or orphans (Gualpa et al. 2019). This had been documented for other rhizosphere bacteria too (Patel et al. 2013). The most recently characterized diazotrophic plant growth promoting *Azospirillum* bacterium, *A. argentinense* type strain Az39 (formerly A. *brasilense*), was isolated from roots of wheat plants in Argentina (Dos Santos Ferreira et al. 2022). It is a plant growth-promoting rhizobacterium, producing IAA besides other phytohormones and harbours several other plant growth-supporting properties including nitrogen fixation (Cassán and Diaz-Zorita 2016). Surprisingly it hydrolyses AHLs, liberating l-HS (Gualpa et al. 2019). Together with the phytohormone-based interaction, the AHL-degradation activity of Az39 may contribute to its strong plant growth-promotion activity, since l-HS has a plant stimulating ability itslef (Palmer et al. 2014). Interestingly, *A. argentinense* Az39 like other strains from the *A. brasilense* cluster (including *A. baldaniorum* Sp245) harbours up to 25 LuxR-proteins (harbouring *LuxR*-solo-sequences) providing receptors for AHLs or related QS-signals from other bacteria or even possibly AHL-unrelated signals from the plant for yet unknown interactions. It is a very efficient bioinoculant for gramineae crops in Argentina (Cassán and Diaz-Zorita 2016). The former species *Azospirillum amazonense* Y1, renamed to *Nitrospirillum amazonense*, carries a canonical *LuxI/R*–QS system and has therefore multiple ways of beneficial interactions with plant hosts based on AHL-signalling. *Nitrospirillum amazonense* strain Y1 is currently used as successful commercial inoculant in sugar cane plantations. Presumably opportunistic human pathogenic bacterial isolates, *Roseomonas fauriae* and *Roseomonas* genomospecies 6, were reported (Cohen et al. 2004), which are phylogenetically very closely related to *A. brasilense* Sp7^T^. However, DNA–DNA hybridization values of 61.2% and 54.2% place these *Roseomonas* isolates into a possibly new species within *Azospirillum* (Hartmann et al. 2019).

The genus *Pseudomonas* harbours a high number of species many of which are associated with diverse plants, having different plant growth-promoting and protecting properties. For example, *P. segetis* P6 isolated from *Salicornia europaea* rhizosphere was characterized to harbour plant growth-promoting activity and QQ-mediated biocontrol (Rodriguez et al. 2020). Seed biopriming of tomato plants with strain P6 resulted in an increase in plant height and weight. Its QQ activity was characterized as an acylase. Thus, strain P6 reduced soft rot symptoms caused by the QS-bacteria *Dickeya solani, Pectobacterium atropsepticum*, and *P. carotovorum* on potato and carrot. The QQ-activities of P6 also protected tomato plants against *P. syringae* pv. tomato, which organizes its virulence through AHL-sensing. Thus, *P. segetis* P6 may have biotechnological applications. *Pseudomonas putida* IsoF, isolated from the rhizosphere of tomato, produces two AHLs, 3-oxo-C10, and 3-oxo-C12 from *ppuI/ppuR* circuit. Interestingly, the Ppu-system controls the expression of a large nonribosomal peptide synthetase, which directs the biosynthesis of two cyclic lipopeptide biosurfactants, putisolvin I and II. Putisolvins inhibit the biofilm formation and also break down existing *P. aeruginosa* biofilms (Cárcamo-Oyarce et al. 2015). A broad range of AHLs and other QS-active compounds are produced by *Pseudomonas* strains, which may have plant growth-promoting but also opportunistic human pathogenic potentials (Venturi 2006). For example, a dominant diazotrophic endophytic *P. aerugionasa* strain PM389 from *Pennisetum glaucum* (L.) was characterized by Gupta et al. (2013). This diazotrophic bacterium even moves upwards to shoots, and revealed various plant growth-promoting properties including mineral phosphate solubilization, siderophore production, and antagonistic biocontrol properties. *Pseudomonas aeruginosa* harbours two complete QS-circuits involving AHL signals and a third system using quinolones, which coordinate virulence acquisition and other behaviours (Miranda et al. 2022). The major AHL 3-oxo-C12-HSL regulates virulence gene expression and also induces mammalian cell responses, including apoptosis and immune modulation (see also below).

*Rhizobium radiobacter* F4 (syn. *Agrobacterium tumefaciens*) was characterized as an endofungal bacterium of *Piriformospora indica* (now known as *Serendipita indica*). *Piriformospora indica* is known as a plant growth-promoting, mycorrhiza-like fungus, able to stimulate growth and performance of many plants especially under biotic and abiotic stress conditions (Varma et al. 2012). Like *P. indica*, the free-living bacterium increases plant biomass and enhances resistance against bacterial leaf pathogens and salt stress (Glaeser et al. 2016). Most interestingly, *R. radiobacter* F4 (RrF4) shows a high degree of similarity to the plant pathogenic *R. radiobacter* C58, formerly named *A. tumefaciens* C58. However, it has important differences in the tumour-inducing plasmid (pTi) lacking the T-region (including the *ipt*-gene) and in the accessory plasmid, because the *virH1*-gene is truncated (Glaeser et al. 2016). This documents, how a plant beneficial bacterium may have developed from a plant pathogenic one. It could be shown that RrF4 produces a spectrum of QS-mediating AHLs with acyl-chains of C8, C10, and C12 as well as hydroxyl- or oxo-sustitutients at the C3-atom (Alabid et al. 2020), which is quite typical for Rhizobia. In *R. radiobacter* F4NM13, a lactonase-overexpressing transconjugant of RfF4, the AHLs were missing and also the plant biomass stimulation as well as the systemic resistance was partially compromised in *Arabidopsis* and wheat. Furthermore, the AHL-deficient transconjugant was lacking cellulose-like fibre scaffolds for efficient root surface attachment. It could not penetrate into the intercellular spaces of the cortex, which is in contrast to the strongly root colonizing endofungal wildtype RrF4 (Alabid et al. 2020). Thus, AHLs contribute to the plant growth stimulation of Rrf4, which may play an important role in the *P. indica* symbiosis with a high diversity of crop and medicinal plants (Varma et al. 2012).

*Acidovorax radicis* N35 is an endophytic bacterium with plant growth-promoting activity in barley and wheat (Li et al. 2011); it was characterized to produce only the QS-autoinducer C10-HSL. An *araI* mutant devoid of C10-HSL production lost the property to efficiently colonize roots of barley. Comparable transcriptome analysis of axenic uninoculated barley seedlings with N35 wild type strain or *araI*-mutant inoculated plants revealed that the mutant-inoculated barley plants accumulated several flavonoids (Han et al. 2016), which can efficiently inhibit root colonization. It may be concluded from this observation that AHL production of the *A. radicis* N35 wild type protect it from plant defence responses of flavonoids.

### Relationship between plant beneficial and human pathogenic bacteria

In the genus *Burkholderia*, harbouring saprophytic, beneficial, symbiotic, human pathogenic, or opportunistic pathogenic species, the assessment of the pathogenic potential of each species was for a long time the reason that regulatory authorities banned all environmental release proposals for any *Burkholderia* strain. The application of whole genome-based comparative software tools together with the assessment of the human pathogenic potential made a clarification possible (Angus et al. 2014). Based on the complete genome sequence data, conserved sequence indels (CSI) were successfully used as molecular marker for the precise identification of three different genera within the *Burkholderia* cluster: *Burkholderia (sensu strictu)*, with pathogenic and opportunistic pathogenic species, *Paraburkholderia*, comprising the plant-associated and -beneficial species (Sawana et al. 2014), and *Caballeronia*, with a group of 12 environmental species (Dobritsa and Samadpour 2016). Several canonical QS-sensing systems mostly based on AHLs and/or also LuxR-solo genes are prevalent in almost all species in the *Burkholderia* cluster, which are the backbone to efficiently organize either virulence or plant health supporting functions.

It is a matter of fact, that plant pathogens can cross the kingdom border and cause human diseases (as reviewed by Kim et al. 2020). In the other direction, some human pathogens, like *Salmonella enterica* typhimurium, are known to have affinity to colonize roots of diverse plants like barley, tomato, or *Arabidopsis*, being even able to colonize the plant hosts endophytically (Kutter et al. 2006, Schikora et al. 2011b, Zarkani et al. 2019). Thus, there are common principles shared by a diversity of bacteria to colonize and enter plant roots as well as mammalian tissues. For example the genus *Herbaspirillum* harbours well-known efficient plant growth-promoting nitrogen-fixing rhizobacteria, but also clinical isolates in *Herbaspirillum* species 3 (Baldani et al. 1996). Strains belonging to *H. seropedicae* and *H. frisingense* (Kirchhof et al. 2001, Straub et al. 2013) were characterized as plant growth-promoting rhizobacteria, but they are also isolated from human skin wounds, sputum samples of cystis fibrosis patients, or other diseased human organs (Faoro et al. 2019, Oliveira et al. 2021). *Herbaspirillum frisingense* Mb11, isolated from roots of the energy plant *Pennisetum purpureum* in Brasil produced AHLs, while the GSF30^T^, derived from *Miscanthus* spp. in Freising, Germany, did not synthesize AHLs; both groups of strains efficiently colonized seedlings of *Miscanthus* and barley endophytically (Rothballer et al. 2008). Within *H. hiltneri*, isolated from surface disinfected wheat roots, which is phylogenetically apart from the *H. seropedica*/*H. frisingense* cluster (Rothballer et al. 2006), nosocomial strains were not yet found. A detailed genomic and proteomic study of clinical and environmental isolates of *H. seropedicae* revealed that clinical strains have lost the gene sets for biological nitrogen fixation (*nif*) and the type 3 secretion system (T3SS), which has been described to be essential for the interaction with plants. A different set of accessory genes and genomic islands could be found in the clinical strains, like genes related to lipopolysaccharide (LPS) biosynthesis and *neuABC* genes, responsible for the biosynthesis of sialic acid. The *neuABC-*linked LPS was able to increase the bacterial resilience in the mammalian host aiding in the escape from the immune system (Faora et al. 2019). In clinical and environmental isolates of *H. frisingense*, the genes in the core and accessory genomes were compared and numerous unique clusters could be identified in clinical and rhizosphere strains. Some genomic islands were only found in clinical strains, while others in all strains. Thus, a preadaptation to different hosts was concluded (Oliveira et al. 2021).

In the case of *Serratia marcescens*, harbouring a wide range of strains from soil, water, and plant surfaces, also opportunistic human pathogens in hospitals and plant growth-promoting bacteria in crops are known. In a pangenome approach, based on available genomic data, whole genome multilocus sequence type schemes (MLSTs) were applied (Abreo and Altier 2019). In most cases, genomes of nosocomial and environmental isolates could be assigned to proposed nosocomial or environmental MLSTs. A minority of nosocomial strains harboured environmental MLSTs, which suggest that these have been recently derived from the environment. One environmental clase had only low numbers of virulence and antibiotic resistance determinants and may represent a group of prospective PGPR strains (Abreo and Altier 2019). In general, it is not entirely clear, whether so-called clinical or nosocomial strains are opportunistic pathogenic bacteria, which cause secondary infections only in immuno-compromised patients and thus worsen conditions in patients. It is a matter of fact that based on general phylogenetic terms, opportunistic pathogenic bacteria can be separated from commensalic and beneficial bacteria only with difficulty. Extensive experimental and genomic assessments of the pathogenic potential of each strain have to be conducted, to allow a case to case decision (Angus et al. 2014; Lee et al. 2014), if the strain should be used as inoculum for plants. A temperature optimum below 37°C is another important criterium for an application in the field. An readdressing of the biosafety level of some already used plant growth promoting rhizobacteria to biosafety level 2 prohibited the continuation of the application in agriculture (Keswani et al. 2019).

### Control of the ecological balance of health supporting and threatening bacteria in mammalian habitats

In comparison to the health situation in the rhizosphere, which is strongly influenced by the plant and the soil microbiome (Hartmann et al. 2009, Schreiter et al. 2014), the microbiological and metabolic quality of food has an important role for the establishment of a balanced structure and function as well as a healthy status of human microbiomes in habitats, such as the oral cavity and the gut. From the early life times as baby and infant, edible plant and other food microbiomes together with a nutritionally balanced food with low fat content and a healthy life style are a good basis for a sustainable health state (Berg et al. 2015, Wassermann et al. 2019, Soto-Giron et al. 2021). It was recently shown that a high diversity of potentially health supporting bacteria are associated with fresh fruits and vegetables harbouring functions for an overall healthy and balanced gut microbiome (Wicaksono et al. 2023). In general, a high diversity of human microbiomes is crucial for persistent health. High fat diet causes a clear shift in the gut microbiome, resulting in a decreased ratio of Bacillota/Actinomycetota (formerly named: Firmicutes/Bacteroidetes) (Daniel et al. 2013, Walker et al. 2014) favouring a disbalanced gut microbiome. Specific sulfonolipids as metabolite markers and related bacterial *Alistipes* and *Odoribacter* species were found specific for this unhealthy situation in the gut of mice (Walker et al. 2017).

### QS-signal production and degradation in the oral cavity

The equilibrium of microbes is maintained through competitive and cooperative interactions. An imbalance of the resident microbiota could be caused by changes in host-dependent habitat conditions or external factors like the quality of food. The development of major oral diseases is usually not dependent on a single oral pathogen, but on the entire microbial community and its activity in the oral cavity (Muras et al. 2020). For example, a reduction of bacterial coaggregation and biofilm formation during dental plaque formation is beneficial for oral and teeth health (Simón-Soro and Mira 2015). Therefore, QS activities, which positively influence biofilm structures, certainly play a major role in caries and peridontal diseases. AI-Ps from Gram-positive bacteria have been identified in different oral streptococci, and AI-2 were frequently found in different Gram-positive and Gram-negative oral pathogens like *Streptococcus mutants* or *Porphyromonas gingivalis* (Frias et al. 2001). Furthermore, different isolates of *Enterobacter* sp., *Pseudomonas* sp., and *Burkholderia* sp. from human tongue surface and dental plaque samples were characterized as AHL-producers (Goh et al. 2014). AHLs could also be detected in saliva and sputum samples indicating a possible role in the dental plaque formation (Goh et al 2014). In addition, QQ-activities were detected in many bacterial isolates from healthy and peridontal patients (Muras et al. 2020). These findings were corroborated by the demonstration that the addition of AHL-lactonase Aii20J had an inhibitory effect on the development of oral biofilms. Using confocal laser scanning microscopy, Aii20J inhibited *in vitro* mixed-species and also saliva biofilms (Muras et al. 2020). Apparently, not only AI-2 inhibitors but also AHL-lactonases or AHL-quenching bacteria are QQ-strategies for the control of oral diseases.

### QQ of bacterial virulence by food substances

Many dietary metabolites were demonstrated to interfere with bacterial virulence and inhibition of QS (Dingeo et al. 2020, Fala et al. 2022). For example, pyrogallol competes with bacterial QS signals for receptor binding, polyphenols and lignans sequester bacterial QS molecules, and the flavanon naringenin reduces the production of QS-controlled virulence in *P. aeruginosa* PAO1. *Capsicum frutescens* is a spicy chilli pepper, which is rich in bioactive compounds such as capsaicin and luteolin. *Capsicum frutescens* extract and pure luteolin-inhibited QS in the model bacterium *Chromobacterium violaceum* and biofilm formation in *P. aeruginosa* PAO1 (Rivera et al. 2019). Apigenein and luteolin are compounds of *Gnapalium hypoleucum* DC extracts showing also strong QS-inhibitory activity (Li et al. 2022). In the model bacterium *C. violaceum* ATCC 12472 *vioB, vioC*, and *vioD* genes were strongly downregulated by apigenin and luteolin. The effective treatment of bacterial infections by *G. hypoleucum* extracts could thus originate from QS inhibition by these natural compounds and provides a potential mechanism for alternative applications of medicinal plants (Li et al. 2022). It was demonstrated that secondary metabolites of medicinal plants, such as terpenoids, flavonoids, and phenolic acids are antibacterial agents. In addition, they exhibit numerous anti-QS mechanisms via the inhibition of autoinducer releases, sequestration of QS-mediated molecules, and deregulation of QS gene expression (Bouyahya et al. 2022).

Microbes in fresh products such as salate or fruits can have a considerable health impact on the gut. Indeed, reconstructed metagenome—assembled genomes from 156 fruits and vegetables revealed that the microbiomes of fresh fruit and vegetables are represented by members of Enterobacteriales, Burkholderiales, and Lactobacillales in the gut microbiome. In these bacterial families diverse QS-signalling activities, but also QQ and quorum-inhibiting microbes and metabolites are represented (Wicaksono et al. 2023).

### QS-related microbe–host interactions in the gut

Functional QS-systems are found not only in pathogenic but also in commensalic gut residents or probiotic bacteria (Fujii et al. 2008). In a healthy situation, gut microbiota mutually interacts with coevolved gut epithelial and immune cells in a beneficial reciprocal way (Coquant et al. 2020). QS-signalling of bacteria was shown to have important roles in beneficial bacteria intestinal cross-talk and contribute substantially to establish cross-kingdom symbiotic interactions (Wu and Luo 2021). In the dysbiosis state, like inflammatory bowel disease (IBD), microbe–intestine interactions drive inflammatory responses. The distribution of AHL-compounds detected in the feces of healthy and IBD-subjects correlated with the disease state (Landman et al. 2018). One of the AHL-compounds, 3-oxo-C12:2-HSL, was highly decreased in fecal samples of IBD patients as compared to remission and healthy persons. Thus, the absence of this particular AHL correlated with dysbiosis. Concomitantly, a decreased level of Firmicutes which are indicative for normobiosis indicated dysbiosis. Furthermore, Landman et al. (2018) showed that 3-oxo-C12:2-HSL exerts anti-inflammatory properties on intestinal model cell lines Caco-2/T17. Therefore, AHL-profiles may be considered as noninvasive biomarkers for gut normobiosis. AI-2, which are produced by opportunistic pathogenic bacteria, were successfully demonstrated *in vivo* to modulate the gut microbiome and cause inflammation (Thompson et al. 2015). ON the other hand, it was demonstrated that mammalian epithelial cells in the gut produce an AI-2 mimicking molecule in response to secreted bacterial factors and tight-junction disruption, which activate QS in bacteria. This was detected by bacterial AI-2 receptors LuxP/LsrB, and by the activation of QS-controlled gene expression of *S. typhimurium* (Ismail et al. 2016). Thus, members of the gut microbiome could be activated to colonize damaged sites and to repair epithelial tight junctions. In mammalian systems the QS-autoinducer 3-oxo-C12-HSL and similar long acylcarbon-chain AHLs produced by *Pseudomonas* and *Burkholderia* spp. are important signalling compounds involved in serious diseases, like COPD. QS-autoinducers are of central importance to coordinate, e.g. biofilm formation and virulence development (Whiteley et al. 2017). While Gram-negative bacteria are known for diverse AHL-production, Gram-positive bacteria, mainly Bacillota and Antinomycetota, are strong players in anti-AHL-QS-quenching systems, possibly acquired by horizontal gene transfer (Rajput and Kumar 2017). QQ-activities of probiotic bacteria were shown to influence the microbial community in the gut as well as immune functions in pigs (Kim et al. 2018). Antagonists of QS-systems of severe pathogens are of great importance, because mechanisms targeting QS have only minor challenge on bacterial cell viability and thus the selection for antibiotic resistant pathogens should be prevented (Zhong and He 2021). In addition, novel screening strategies were developed for QS-inhibitors to combat bacterial infections (Lu et al. 2022).

QS-signalling can organize competing strategies to neighbouring microbes. For example Gram-positive Propionibacteria, like *Propionibacterium freudenreichii*, have promising probiotic properties (Rabah et al. 2017, Savijoki et al. 2023). Progress in defining such metabolic interactions by *in situ* screening test was made possible by using biofilm-forming *C. violaceum* as a QS-reporter and a microscale screening platform. In this way, anti-QS effects of *Lactobacillus acidophilus, Lacticaseinbacíllus rhamnosus, P. freudenreichii*, and other cheese-associated strains could be identified as potent competitors for virulent pathogens (Savijoki et al. 2023).

### Mechanisms of signal perception

Similar to the plant innate immune system, pathogen-associated molecular patterns are recognized by highly sensitive and specific recognition receptors such as the toll-like receptors in mammals. In addition, the adaptive immune system with differentiating dendritic cells (DC) and a guild of T-cells and macrophages are active in a coordinated way building up the most efficient adaptive immune response of mammals. Furthermore, specific receptors are involved for bacterial sensing and perception by the mammalian/human system, including QS-associated molecules and activities (Holm and Vikstrom 2014). In this context, numerous *LuxR-*solo type orphan genes in the human genome may code for receptors responding to bacterial products, including QS-compounds, but also for unrelated metabolites of the host or other members of complex holobiont in a still unknown way (Yong and Zhong 2013, Uhlig and Hyland 2022). The production of short chain fatty acids (SCFA) produced by commensal bacteria and probiotics in the gastrointestinal tract (GT) contribute to a balanced situation by stimulating e.g. the bacteriocin production of probiotic *Lactobacillus* strains, which control the virulence status of pathogens (Meng et al. 2021).

G protein-coupled receptors (GPCRs) are potential targets facilitating bacteria–host interactions by microbial-derived molecules including QS-signals. This was shown for the stimulation of root growth by AHLs with C6- and C8-fatty acid chain lengths in *A. thaliana* (von Rad et al. 2008, Liu et al. 2012) (see above) In general, GPCRs are members of a frequently found gene family in the plant and human genomes, some of which are involved in bacterial sensing (Krasulova and Illes 2021). Other non-GPCR targets were also described in sensing bacterial QS signals.

In mammalian systems, an uptake of 3-oxo-C12-HSL was shown in experimental human lung epithelial cells (Bryan et al. 2010). An microarray of transcriptional responses of lung epithelial cells after exposure to 3-oxo-C12-HSL revealed the expression of several xenobiotic-sensing and drug transport genes. Using radiolabelled autoinducer uptake assays, increased intracellular 3-oxo-C12-HSL levels were found after exposure, which decreased afterwards to background levels. Since this process was inhibited by the ABC transporter ABCA1, it was concluded that mammalian cells detect and take up 3-oxo-C12-HSL, but expel it later after activation of protective transport systems (Bryan et al. 2010).

AHLs were recognized as activators of the cytosolic aryl-hydrocarbon receptors (AhRs), which respond to plant products, xenobiotics, indole molecules, and SCFAs. AhR-activity is differently regulated by distinct QS molecules (Sun et al. 2020), which could constitute a crucial role of AhR in the regulation of host metabolism by pathogenic, commensal, and probiotic bacteria (Karlsson et al. 2012, Natividad et al. 2018). Interestingly, d-tryptophan (d-Trp) was identified as excreted metabolite of several probiotic bacteria, including *L. rhamnosus* GG, which may interact with the AhR-receptor (Kepert et al. 2017). d-Trp was shown to have stimulatory functions on developing DC-cells, to exert antiallergic responses, and to modify the gut microbiome of mice (Kepert et al. 2017). In addition to known receptor-type sensors, the lipid composition of the cell membrane, and the expression of glycolipids and transmembrane proteins were recently suggested to modulate the perception of QS and other signals and their perception (Uhlig and Hyland 2022).

Bacteria defective in QS signalling are less efficient in colonizing the GT, which was found in commensal, probiotic, and pathogenic strains (Whiteley et al. 2017). For example, *Streptococcus gallolyticus* subsp. *gallolyticus* can only colonize the murine intestine when the QS-regulated bacteriocine-like peptides blpA and blpB are produced, since Blp-deficient mutants could not sustain in the intestine. LuxS-signalling of *L. rhamnosus* GG and *Bifidobacterium breve* UCC2003 is required for the adhesion to intestinal cells (Jiang et al. 2021) and persistence in the gastro-intestinal (GI) tract (Christiaen et al. 2014).

### Interactions with the human immune system

**Figure 3.**
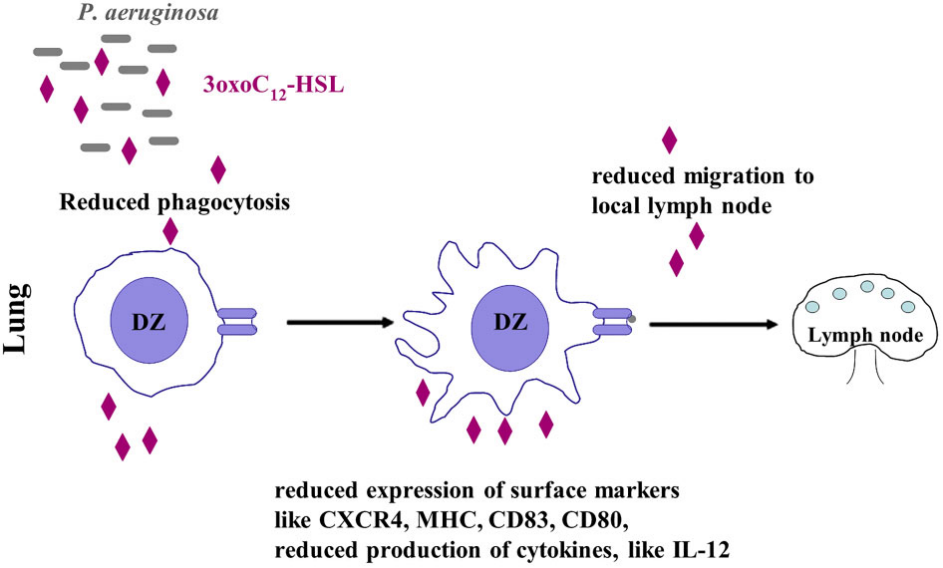
AHL-interactions with mammalian immune system (example: 3-oxo-C12-HSL of *P. aeruginosa* and lung) (Binder, PhD thesis, LMU Munich 2010): DC are influenced by 3-oxo-C12-HSL during their ripening process stimulated by lipopolysacharide (LPS), leading to diminished phagocytosis and reduced expression of surface markers, like CXCR4, MHC, CD83, and CD80. The CXCR4-marker is involved in the regulation of migration of DC to lymph nodes, while the reduction of other surface markers lead to the induction of cyclooxygenase (Cox-2) having a modulatory role in inflammation processes; prostaglandines are increased supporting the Th2-response in the lymph node, which support a reduced ripening of DC-cells, meaning reduced inflammation response (Skindersoe et al. 2009). Downregulation of cytokines, like IL-12 and the anti-inflammatory cytokine IL-10, causes a Th1/Th2-imbalance in the infected host (Ritchie et al. 2005).

Concerning responses of gut microbiota to host metabolites, Gram-negative bacteria *Escherichia coli, Shigella* sp., and *Salmonella* sp. express QseC, a membrane-bound histidine sensor kinase that allows them to react to host-stress signals such as epinephrine and norepinephrine. Qsec inhibitors were developed as antivirulence approach against Gram-negative diseases (Rasko and Sperandio 2010, Curtis and Russell 2014). This interkingdom activation is referred to the autoinducer-3 (AI-3) system leading to the activation of QS gene expression. QseC sensor kinase is involved in GI disease caused by *E. coli* pathogens in rabbits. The habitat for direct interference of gut microbiota and human host are the inner mucus and the gut epithelial barrier. The structure of the intestinal mucus and epithelial barrier functions are greatly influenced by the host defence status and the QS-activity of the gut microbiome. While probiotics enhance barrier functions *in vitro* and in animal studies, QS-regulated virulence factors from pathogenic *Citrobacterium difficile, E. coli*, and *S. typhimurium* decrease transepithelial resistance by modulation tight barrier junctions. In contrast, QS-associated activities, involving 3-oxo-C12 HSL, were found to negatively impact gut integrity through the activation of inflammatory pathways that impair intestinal barrier function (Adiliaghdam et al. 2019). AHLs, mostly 3-oxo-C12- HSL from *P. aeruginosa*, stimulate inflammatory responses through the inhibitory interaction with neutrophils, macrophages, and DC, finally causing apoptosis of those immune cells. In this way, 3-oxo-C12-HSL producing bacteria pathogens effectively inhibits attempts oft he immune system to eliminate the aggressor (Coquant et al. 2020). When LPS-stimulated human DC were exposed to 3-oxo-C12-HSL, *in vitro* flow cell cytometric analyses revealed that important DC surface markers like CD80, CD40, CD184, and HLA-DR were diminished, while 3-oxo-C4-HSL had no effect (Binder T, PhD thesis LMU Munich, 2010). Accordingly, the inflammation inhibitory cytokine IL-10 was decreased and the inflammation stimulatory cytokine IL-8 was increased, leading to a severe damping effect on the immune response. Furthermore, the migration of DC-cells after treatment with LPS and 3-oxo-C12-HSL was decreased as well as their phagocytotic activity. This causes severe health problems of cystic fibrosis patients infected with *P. aeruginosa* (Cohen et al. 2015). Figure 3 shows a schematic drawing of activities of the autoinducer 3-oxo-C12-HSL of *P. aeruginosa* towards the human innate and adaptive immune system.

## Conclusions

Microbial health in the rhizosphere is interconnected with ‘One Health’, because the health of each of its components is determined by the omnipresence of microorganisms (Banerjee et al. 2023). Rhizosphere microbiomes are tightly linked to soil and plant microbiomes and their reservoirs for extremely diverse microbioms also influence animal and human health (Hartmann et al. 2009, Schreiter et al. 2014). In the rhizosphere, a rich supply of substrates leaking out of roots guarantees excellent conditions for microbial activities, which also provide the basis for efficient coevolution of environmental and plant microbes with each other in the context of the plant host as master. This includes natural genetic engineering using horizontal gene transfer, plasmid transduction, high mutation rates, and phenotypic switching. In the efficient ‘rhizosphere schools’ of evolution the selection of adapted rhizosphere communities through changing the exudation pattern constantly occurs (Berendsen et al. 2012). Under these selective conditions QS-signalling-based microbe–host interactions towards beneficial/cooperative as well as pathogenic/competitive relations have evolved. The knowledge about responses of plant and mammalian/human hosts to bacterial QS-signalling compounds and their producers are considerable advanced. A large body of data about the interaction of pathogenic, symbiotic/beneficial, and commensalic bacteria accumulated, which are involved in plant or human health (LaSarre and Federle 2013, Mendes et al. 2013, Schikora et al. 2016). In this context, QS research provides new perspectives to better understand the interaction between gut microbiota and the human host (Yang et al. 2022). This may provide an additional key to be able to apply microbiome science for plant and human health (Russ et al. 2023). Nowadays, first experiences about the possibility of translation into practice with recognized specific ‘therapeutic’ microbes and QS-related substances (Uhlig and Hyland 2022), QS-signals (Moshynets et al. 2019), and synthetic microbial communities (syn-coms) (Jiang et al. 2022, Schmitz et al. 2022) are available. Apparently, an effective syn-com may harbour QS-active bacteria, which provide the applied synthetic community a dynamic character to modulate the rhizosphere microbiome and stimulate the plant host to develop abiotic and biotic resistance properties (Andres-Barrao et al. 2017). Further progress in revealing the involvement of QS-regulation in plant as well as in human health is still dependent on further optimization of the sensitivity and specificity of QS-signal analytical approaches (Mellini et al. 2024). Concerning the severe problem of rapid spreading of chronic infections in humans because of the immense dangerous threat by multiresistant pathogens, the application of QS-affecting approaches, which target virulence acquisition processes of pathogens promised to avoid the selection of antibiotic resistances, because growth of pathogens is not directly targeted. This could finally lead to substantial human health improvements in the control of multiresistant pathogens when combined with other strategies (Zhong and He 2021, Naga et al. 2023). However, since QS-systems are important for bacterial fitness, QS-inhibition would unavoidingly affect the targeted pathogens and, may hence impose a selective pressure. Experimental evidences for this problem was published by Maeda et al. (2012) and Imperi et al. (2019). Thus, strategies based on system-level ecologic principles of microbial social behaviour may contribute to successful personalized treatments of patients. Applications of QS-targeted treatments could allow important progress to improve sustainable agriculture and contribute to better control devastating diseases for plant and human health.
